# Pituitary hypoadrenocorticism and hypothyroidism after immunochemotherapy followed by salvage surgery for lung cancer: a case report

**DOI:** 10.1186/s44215-022-00019-w

**Published:** 2023-03-16

**Authors:** Chikara Nakagami, Makoto Endoh, Marina Nakatsuka, Kenta Nakahashi, Hiroki Ota, Mari Aso, Takuma Sugiyama, Hiroki Suzuki, Satoshi Shiono

**Affiliations:** 1 Department of Thoracic Surgery, Yamagata Prefectural Central Hospital, 1800 Ooazaaoyagi, Yamagata-shi, Yamagata, 990-2292 Japan; 2 Department of Respiratory Medicine, Yamagata Prefectural Central Hospital, 1800 Ooazaaoyagi, Yamagata-shi, Yamagata, 990-2292 Japan; 3 Department of Diabetes and Endocrinology, Yamagata Prefectural Central Hospital, 1800 Ooazaaoyagi, Yamagata-shi, Yamagata, 990-2292 Japan; 4 Department of Surgery II, Faculty of Medicine, Yamagata University, 2-2-2 Iida-Nishi, Yamagata-shi, Yamagata, 990-9585 Japan

**Keywords:** Lung cancer, Surgery, Immune checkpoint inhibitor, Immunochemotherapy, Immune-related adverse events

## Abstract

**Background:**

Immune checkpoint inhibitors (ICIs) have been shown to prolong the survival of patients with non-small cell lung cancer (NSCLC) and have allowed complete resection for advanced lung cancer. However, immune-related adverse events (irAEs) have been recognized as concerning side effects of ICIs.

**Case presentation:**

A 62-year-old man visited our hospital because of fever, dyspnea, and anorexia. A tumor was found in the right hilum of the lung. It compressed the left atrium and was also thought to be invading the esophagus and a vertebral body. A bronchoscopic biopsy revealed squamous cell carcinoma of the lung (cT4N2M0-IIIB). We thought that a complete resection was impossible because of the N2 status of the tumor and because it had invaded several organs. Radiotherapy was thought to be contraindicated because of the patient’s marked emphysema. Therefore, we administered 4 courses of pembrolizumab plus carboplatin plus nab-paclitaxel immunochemotherapy.

After immunochemotherapy, the tumor was downstaged to ycT2bN0M0-IIA and was determined to be acceptable for salvage surgery. A right lower lobectomy and systematic dissection of the mediastinal lymph nodes were performed. The histopathological examination of the resected specimen found that the proportion of the remaining tumor cells was 5%, indicating achievement of a major pathologic response.

On postoperative day 79, the patient visited the emergency room because of anorexia. Blood tests showed hyponatremia, hypoglycemia, and eosinophilia. The serum thyroid hormone and thyroid-stimulating hormone levels were low and high, respectively. A corticotropin-releasing hormone stimulation test revealed levels of adrenocorticotropic hormone and cortisol far below the normal ranges. We speculated that the patient had developed pituitary hypoadrenocorticism and hypothyroidism as irAEs associated with ICI treatment*.* We administered hydrocortisone and levothyroxine, with improvement in the patient’s appetite and normalization of the patient’s serum sodium level. The patient has been receiving ongoing supplementation with oral hydrocortisone and levothyroxine and is doing well 11 months after surgery.

**Conclusions:**

The increasing numbers of patients treated with perioperative ICIs might lead to increasing numbers of patients who develop perioperative irAEs. Careful attention should be paid to the possible development of irAEs during the perioperative management of patients undergoing surgery for lung cancer.

**Supplementary Information:**

The online version contains supplementary material available at 10.1186/s44215-022-00019-w.

## Background

Immune checkpoint inhibitors (ICIs) have been shown to contribute to the prolonged survival of patients with non-small cell lung cancer (NSCLC) and have enabled the option of performing complete resections of advanced lung cancers [1–8]. However, the administration of ICIs has been found to be associated with the development of immune-related adverse events (irAEs) [9–11]. Additionally, few reports have been published on the occurrence of irAEs in patients who had received immunochemotherapy followed by salvage surgery for NSCLC.

Here, we report a patient with lung cancer who underwent immunochemotherapy followed by surgery and subsequently presented with the irAEs of pituitary hypoadrenocorticism and hypothyroidism.

## Case presentation

**Fig. 1:**
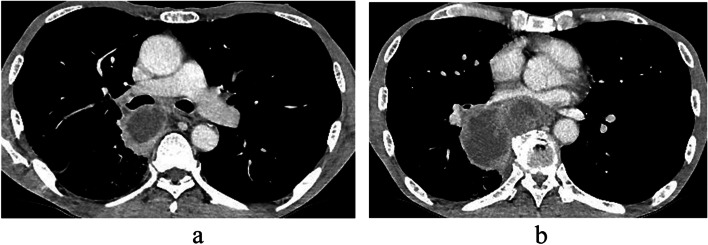
Preoperative computed tomography images. The tumor was 73 mm in diameter and located in the right hilum of the lung (**a**). It compressed the left atrium of the heart. It appeared to invade the esophagus and a vertebral body (**b**)

A 62-year-old man was admitted to our hospital because of fever, dyspnea, and anorexia. His medical history was insignificant except for a 44 pack-year history of smoking. His performance status (PS) was 1, and he had an elevated serum level of carcinoembryonic antigen (5.56 ng/mL). Chest computed tomography (CT) revealed a 73 mm tumor in the hilum of the right lung that compressed the left atrium. The tumor was also suspected of invading the esophagus and a vertebral body (Fig. 1).

**Fig. 2:**
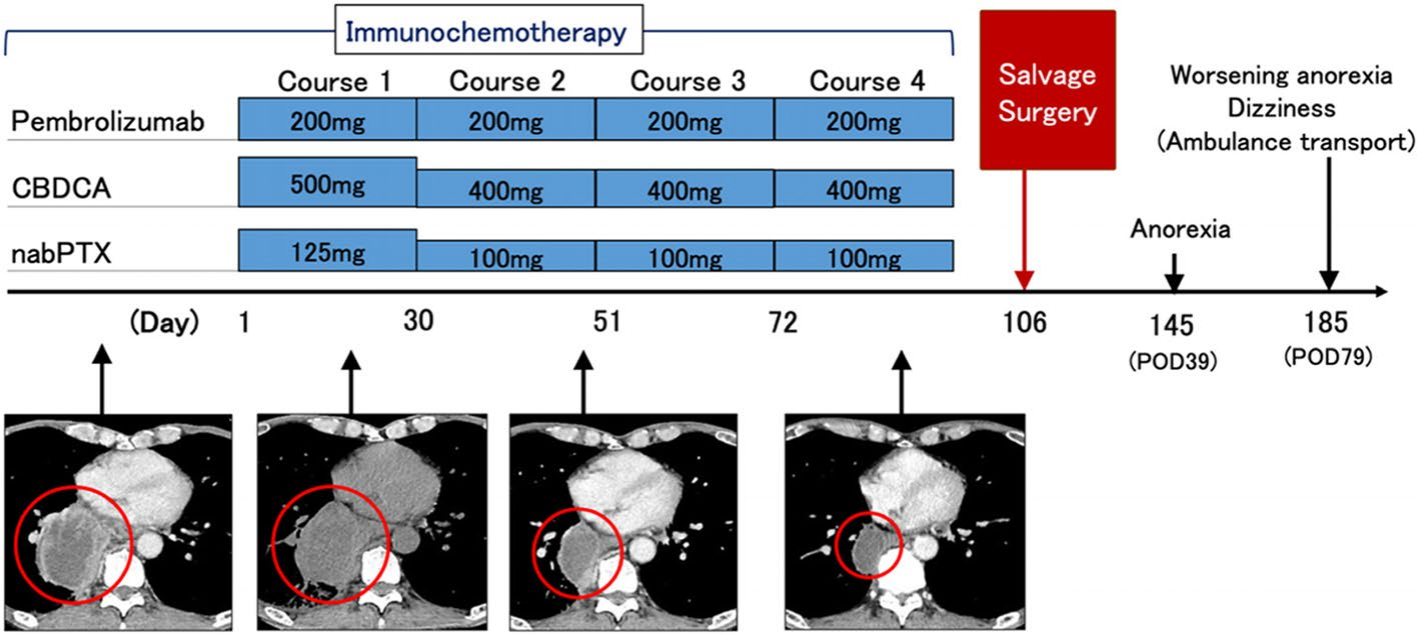
Clinical course of the patient: four courses of pembrolizumab and carboplatin (CBDCA) plus nabPTX) chemotherapy were administered. After one course, because the patient developed pneumonia, the amounts of the CBDCA and nabPTX were reduced. After the treatment, CT showed that the tumor shrank (images of tumor are identified by red circles), which was considered to be a partial response. Radical surgery was performed on day 34 from the day of the last (fourth) administration of pembrolizumab. On postoperative day 39, the patient complained of anorexia, and on postoperative day 79, he went to the emergency room because of dizziness and worsening anorexia

Examination of an endobronchial ultrasound-guided transbronchial needle aspirate from the subcarinal lymph node found squamous cell carcinoma of the lung. There were no distant metastases, and the patient was diagnosed with cT4N2M0-stage IIIB squamous cell carcinoma of the lung. Because the tumor was N2, and was suspected of invading the esophagus, a vertebral body, and left atrium of the heart, complete resection was thought to be impossible. In addition, he had marked emphysema due to smoking, which made radiotherapy difficult. Since the tumor cells were programmed cell death ligand 1 (PD-L1) positive (tumor proportion score = 1%), the patient underwent 4 courses of pembrolizumab plus carboplatin plus nab-paclitaxel chemotherapy in accordance with treatment guidelines for stage 4 NSCLC (Fig. 2).

Posttreatment CT showed that the tumor had decreased to 44 mm in diameter, which was a partial response. The tumor was downstaged to ycT2bN0M0-stage 2A, which was considered to be resectable by radical surgery. The patient’s preoperative parameters were as follows: vital capacity, 1.92 L; forced expiratory volume in one second, 0.79 L; ratio of forced expiratory volume in one second/forced vital capacity, 43.2%; diffusing capacity for carbon monoxide, 6.40 mL/min/mm Hg; and % diffusing capacity for carbon monoxide, 51.2%. Although the patient’s respiratory function was reduced, he was young, and his PS had improved to 0, therefore, he underwent radical surgery 4 months after receiving immunochemotherapy.

A right lower lobectomy and systematic mediastinal lymph node dissection (ND2a-2) were performed (Additional file 1). The histopathological diagnosis was a keratinizing squamous cell carcinoma with a 4 × 4 mm residual tumor. The proportion of residual tumor cells was 5%, which was a major pathologic response (ypT1miN0M0-stage 1A1). The postoperative course was uneventful, and the patient was discharged on postoperative day 6.

On postoperative day 39, he complained of anorexia, and on postoperative day 79, he came to the emergency room because of dizziness and worsening anorexia. Blood testing showed hyponatremia, hypoglycemia, and eosinophilia (Table 1), which suggested endocrine disorders. He underwent further assessments of his endocrine system. His free triiodothyronine (T3), thyroxine (T4), and adrenocorticotropic hormone (ACTH) serum levels were low, and his serum thyroid-stimulating hormone (TSH) level was high (Table 1). Magnetic resonance imaging revealed swelling of the pituitary gland. A corticotropin-releasing hormone stimulation test showed that the levels of ACTH and cortisol were far below the normal ranges. We concluded that the patient had concomitant pituitary hypoadrenocorticism and hypothyroidism.

**Table 1:** Laboratory data on postoperative day 79. Blood testing showed hyponatremia, hypoglycemia, and eosinophilia. The free T3 and T4 levels were decreased, and the TSH level was elevated. The ACTH level was also decreased

Peripheral blood	
Hb	11.2 g/dL
WBC counts	7.56 × 10^3^/μL
Neut	77.20%
Eosino	6.70%
Baso	0.40%
Mono	4.50%
Lymph	11.20%
Plt counts	5.55 × 10^5^/μL
Biochemistry	
Plasma glucose	70 mg/dL
HbA1c	6.30%
Free T3	< 0.95 pg/mL
Free T4	< 0.42 ng/mL
TSH	171 μIU/mL
Plasma renin activity	0.4 ng/mL/h
Plasma aldosterone concentration	183 pg/mL
Growth hormone	4.64 ng/mL
Insulin-like growth factor 1	23 ng/mL
ACTH	< 1.5 pg/mL
Antidiuretic hormone	3.4 pg/mL
Cortisol	9.28 μg/dL
Dehydroepiandrosterone sulfate	16 μg/dL
Luteinizing hormone	4.08 mIU/mL
Follicle-stimulating hormone	7.00 mIU/mL
Prolactin	48.2 ng/mL

Oral and intravenous hydrocortisone were started, followed by oral levothyroxine. Specifically, the patient started taking oral hydrocortisone (15 mg/day) on the day of admission (postoperative day 79). Hydrocortisone was additionally administered either intravenously or orally from days 2 to 9 of hospitalization. Intravenous hydrocortisone (100 mg/day) was administered on days 2 to 4 of hospitalization followed by 75 mg/day on day 5 and 50 mg/day on day 6. The patient received oral hydrocortisone (15 mg/day) on hospitalization days 7 to 9.

On day 7 of hospitalization, he also started to receive oral levothyroxine (25 μg/day), followed by 50 μg/day on days 13 to 19, and thereafter starting on day 20, he received 75 μg/day.

The patient’s appetite improved on day 4 of hospitalization and his serum sodium level increased and normalized on day 11 of hospitalization. The patient was discharged on day 22 of hospitalization. The patient has been receiving ongoing supplementation with oral hydrocortisone and levothyroxine and is doing well 11 months after surgery.

## Discussion

According to several global guidelines for patients with unresectable stage 3 NSCLC, cisplatin-based chemoradiotherapy followed by durvalumab is a standard practice [12, 13]. However, when neither radical surgery nor radiotherapy is possible, as in our patient, the guidelines recommend administering chemotherapy based on stage 4 NSCLC. Because the tumor cells were PD-L1 positive, the patient received pembrolizumab plus carboplatin plus nab-paclitaxel chemotherapy, which is the gold standard therapy as recommended by several global guidelines [12, 13].

At present, there are several ongoing clinical trials on preoperative ICIs. Published studies have already reported promising results for obtaining complete resections in patients with advanced lung cancer [1–3]. Phase 2 clinical trials such as the NADIM (NCT02716038) [4] and NEOSTAR (NCT03158129) [5] trials showed that preoperative immunochemotherapy obtained increased rates of major pathologic response or complete pathologic response. Other trials that have included patients with N2-IIIB lung cancer, such as the LCMC3 (NCT02927301) and KEYNOTE-671 (NCT03425643) trials, are in progress [6].

IrAEs associated with ICIs have become a concern [9–11]. These irAEs are completely different from the AEs associated with conventional anticancer agents. The most frequently reported AEs associated with ICIs involve the skin, gastrointestinal tract, liver, endocrine system, and lungs [14–16]. The KEYNOTE-407 study of pembrolizumab plus chemotherapy for advanced and relapsed squamous cell carcinoma of the lung found that 45 of 278 patients (16.2%) developed thyroid dysfunction, with 3 patients (1.1%) showing grade 3 or higher dysfunction. Pituitary dysfunction was observed in 3 patients (1.1%), with 2 patients (0.7%) showing grade 3 or higher dysfunction. Adrenal insufficiency was not observed in any patient [7].

The clinical trials that have investigated the preoperative ICIs for patients with NSCLC have not reported the rates of irAEs in their patients. Although irAEs could have developed during neoadjuvant therapy in the clinical trials setting, few reports have detailed documentation on the time of onset of irAEs. There are also few reports on the occurrence of irAEs after surgery for NSCLC.

The most common reported side effects associated with the administration of pembrolizumab to patients with NSCLC were hypo- and hyperthyroidism [17]. Although pembrolizumab plus a platinum-based regimen were reportedly less likely to lead to an endocrine disorder than other immunochemotherapies such as nivolumab, durvalumab, and atezolizumab [18], whether there are differences between the rates of irAEs associated with various ICIs has not been clarified. Anti-PD-L1 agents have produced a higher incidence of thyroid dysfunction compared to anticytotoxic T lymphocyte antigen-4 antibodies [19, 20].

Our patient had both hypothyroidism and secondary hypoadrenocorticism due to hypopituitarism. In addition to the AEs associated with immunochemotherapy, it cannot be ruled out that the stress of the surgery may have adversely affected the patient’s endocrine function.

Surgical stress can inhibit the peripheral conversion of T4 to T3, which can induce a hypothyroid-like state [21]. Furthermore, stressful events may also precipitate hypopituitarism [22]. We should keep in mind that endocrine abnormalities can occur during the perioperative period in patients who have received immunochemotherapy. Measurements of cortisol and free T3 and T4 levels during the perioperative and postoperative periods should be useful for the early detection of endocrine abnormalities.

Corticosteroids are used to treat hypoadrenocorticism resulting from the decreased secretion of ACTH due to hypopituitarism [23]. Levothyroxine, a thyroid hormone, is used to treat hypothyroidism. In patients with hypothyroidism concomitant with hypoadrenocorticism, corticosteroids should always be administered first, which can lead to acute adrenal crisis.

Perioperative irAEs associated with ICIs have problems. Firstly, the times to onset of irAEs remain unclear. Sun et al. reported that the median time from the start of the administration of ICIs to the onset of irAEs was 10 weeks, and the interquartile range of 6–19.5 weeks was relatively wide [24].

Secondly, patients with irAEs present with various signs/symptoms, including headache, visual abnormalities, tachycardia, fatigue, muscle pain, weight changes, dizziness, and dry mouth [23]. When patients present with such signs/symptoms after lung cancer surgery, irAEs must be differentiated from non-irAEs. Furthermore, based on our patient and another report, periodic endocrinological testing is also warranted when ICIs are administered perioperatively [23].

In conclusion, we reported a rare case of postoperative irAEs that developed after the administration of ICI followed by salvage surgery. As the number of patients treated with perioperative ICIs increases, the number of patients developing perioperative irAEs might also increase. Careful attention should be paid to the possible development of irAEs during the perioperative management of patients undergoing surgery for lung cancer.

## Supplementary Information

**Additional file 1.** Intraoperative video.

## Data Availability

All data supporting this study are included in this manuscript.

## Abbreviations

ICIs: Immune checkpoint inhibitors
NSCLC: Non-small cell lung cancer
irAEs: Immune-related adverse events
CT: Computed tomography
ACTH: Adrenocorticotropic hormone
TSH: Thyroid-stimulating hormone
T3: Triiodothyronine
T4: Thyroxine

## References

[1] Forde PM, Chaft JE, Smith KN, Anagnostou V, Cottrell TR, Hellmann MD, et al. Neoadjuvant PD-1 blockade in resectable lung cancer. N Engl J Med. 2018;378:1976–86. 29658848 10.1056/NEJMoa1716078 PMC6223617

[2] Soh J, Hamada A, Fujino T, Mitsudomi T. Perioperative therapy for non-small cell lung cancer with immune checkpoint inhibitors. Cancers (Basel). 2021;13:4035. 34439189 10.3390/cancers13164035 PMC8391213

[3] Yamaguchi M, Nakagawa K, Suzuki K, Takamochi K, Ito H, Okami J, et al. Surgical challenges in multimodal treatment of N2-stage IIIA non-small cell lung cancer. Jpn J Clin Oncol. 2021;51:333–44. 33506253 10.1093/jjco/hyaa249

[4] Provencio M, Nadal E, Insa A, García-Campelo MR, Casal-Rubio J, Dómine M, et al. Neoadjuvant chemotherapy and nivolumab in resectable non-small-cell lung cancer (NADIM): an open-label, multicentre, single-arm, phase 2 trial. Lancet Oncol. 2020;21:1413–22. 32979984 10.1016/S1470-2045(20)30453-8

[5] Cascone T, William WN Jr, Weissferdt A, Leung CH, Lin HY, Pataer A, et al. Neoadjuvant nivolumab or nivolumab plus ipilimumab in operable non-small cell lung cancer: the phase 2 randomized NEOSTAR trial. Nat Med. 2021;27:504–14. 33603241 10.1038/s41591-020-01224-2 PMC8818318

[6] Yang ZR, Liu MN, Yu JH, Yang YH, Chen TX, Han YC, et al. Treatment of stage III non-small cell lung cancer in the era of immunotherapy: pathological complete response to neoadjuvant pembrolizumab and chemotherapy. Transl Lung Cancer Res. 2020;9:2059–73. 33209626 10.21037/tlcr-20-896 PMC7653116

[7] Paz-Ares L, Vicente D, Tafreshi A, Robinson A, Soto Parra H, Mazières J, et al. A randomized, placebo-controlled trial of pembrolizumab plus chemotherapy in patients with metastatic squamous NSCLC: protocol-specified final analysis of KEYNOTE-407. J Thorac Oncol. 2020;15:1657–69. 32599071 10.1016/j.jtho.2020.06.015

[8] Shen D, Wang J, Wu J, Chen S, Li J, Liu J, et al. Neoadjuvant pembrolizumab with chemotherapy for the treatment of stage IIB-IIIB resectable lung squamous cell carcinoma. J Thorac Dis. 2021;13:1760–8. 33841966 10.21037/jtd-21-103 PMC8024839

[9] Chang LS, Barroso-Sousa R, Tolaney SM, Hodi FS, Kaiser UB, Min L. Endocrine toxicity of cancer immunotherapy targeting immune checkpoints. Endocr Rev. 2019;40:17–65. 30184160 10.1210/er.2018-00006 PMC6270990

[10] Okura N, Asano M, Uchino J, Morimoto Y, Iwasaku M, Kaneko Y, et al. Endocrinopathies associated with immune checkpoint inhibitor cancer treatment: a review. J Clin Med. 2020;9:2033. 32610470 10.3390/jcm9072033 PMC7409155

[11] Postow MA, Sidlow R, Hellmann MD. Immune-related adverse events associated with immune checkpoint blockade. N Engl J Med. 2018;378:158–68. 29320654 10.1056/NEJMra1703481

[12] National Comprehensive Cancer Network. NCCN Clinical Practice Guidelines in Oncology: non-small cell lung cancer. Version 4. 2022. https://www.nccn.org/professionals/physician_gls/pdf/nscl.pdf. Accessed 2 Sept 2022.

[13] Postmus PE, Kerr KM, Oudkerk M, Senan S, Waller DA, Vansteenkiste J, et al. Early and locally advanced non-small-cell lung cancer (NSCLC): ESMO Clinical Practice Guidelines for diagnosis, treatment and follow-up. Ann Oncol. 2017;28(suppl 4):iv1–21. 28881918 10.1093/annonc/mdx222

[14] Postow MA. Managing immune checkpoint-blocking antibody side effects. Am Soc Clin Oncol Educ Book. 2015:76–83. 10.14694/EdBook_AM.2015.35.76. 10.14694/EdBook_AM.2015.35.76 25993145

[15] Davies M, Duffield EA. Safety of checkpoint inhibitors for cancer treatment: strategies for patient monitoring and management of immune-mediated adverse events. Immunotargets Ther. 2017;6:51–71. 28894725 10.2147/ITT.S141577 PMC5584920

[16] Weber JS, Hodi FS, Wolchok JD, Topalian SL, Schadendorf D, Larkin J, et al. Safety profile of nivolumab monotherapy: a pooled analysis of patients with advanced melanoma. J Clin Oncol. 2017;35:785–92. 28068177 10.1200/JCO.2015.66.1389

[17] Wang PF, Chen Y, Song SY, Wang TJ, Ji WJ, Li SW, et al. Immune-related adverse events associated with anti-PD-1/PD-L1 treatment for malignancies: a meta-analysis. Front Pharmacol. 2017;8:730. 29093678 10.3389/fphar.2017.00730 PMC5651530

[18] Zhang W, Gu J, Bian C, Huang G. Immune-related adverse events associated with immune checkpoint inhibitors for advanced non-small cell lung cancer: a network meta-analysis of randomized clinical trials. Front Pharmacol. 2021;12:686876. 34759817 10.3389/fphar.2021.686876 PMC8574003

[19] de Filette J, Andreescu CE, Cools F, Bravenboer B, Velkeniers B. A systematic review and meta-analysis of endocrine-related adverse events associated with immune checkpoint inhibitors. Horm Metab Res. 2019;51:145–56. 30861560 10.1055/a-0843-3366

[20] Anderson B, Morganstein DL. Endocrine toxicity of cancer immunotherapy: clinical challenges. Endocr Connect. 2021;10:R116–24. 33544091 10.1530/EC-20-0489 PMC8052567

[21] Beauchamp RD, Evers BM, Mattox KL, Townsend CM. Sabiston Textbook of Surgery. 19th ed. Amsterdam: Elsevier Health Sciences; 2012.

[22] Hahner S, Loeffler M, Bleicken B, Drechsler C, Milovanovic D, Fassnacht M, et al. Epidemiology of adrenal crisis in chronic adrenal insufficiency: the need for new prevention strategies. Eur J Endocrinol. 2010;162:597–602. 19955259 10.1530/EJE-09-0884

[23] Brahmer JR, Lacchetti C, Schneider BJ, Atkins MB, Brassil KJ, Caterino JM, et al. Management of immune-related adverse events in patients treated with immune checkpoint inhibitor therapy: American society Of Clinical Oncology Clinical Practice Guideline. J Clin Oncol. 2018;36:1714–68. 29442540 10.1200/JCO.2017.77.6385 PMC6481621

[24] Sun X, Roudi R, Dai T, Chen S, Fan B, Li H, et al. Immune-related adverse events associated with programmed cell death protein-1 and programmed cell death ligand 1 inhibitors for non-small cell lung cancer: a PRISMA systematic review and meta-analysis. BMC Cancer. 2019;19:558. 31182061 10.1186/s12885-019-5701-6 PMC6558759

